# Whole-genome sequencing and analysis of *Chryseobacterium arthrosphaerae* from *Rana nigromaculata*

**DOI:** 10.1186/s12866-024-03223-6

**Published:** 2024-03-08

**Authors:** Lihong Zhu, Hao Liu, Xiaohui Li, Yuefeng Shi, Xiaoliang Yin, Xionge Pi

**Affiliations:** 1 Institute of Plant Protection and Microbiology, Zhejiang Academy of Agricultural Sciences, Hangzhou, 310021 China; 2 College of Bioengineering, Tianjin University of Science and Technology, Tianjin, 300457 China; 3 Business Integrated Services Center of Donghu Town, Shaoxing City in Zhejiang Province, Zhejiang Shaoxing, 312001 China

**Keywords:** *Rana nigromaculata*, *Chryseobacterium arthrosphaerae*, Whole-genome sequence, Bioinformatics analysis

## Abstract

*Chryseobacterium arthrosphaerae* strain FS91703 was isolated from *Rana nigromaculata* in our previous study. To investigate the genomic characteristics, pathogenicity-related genes, antimicrobial resistance, and phylogenetic relationship of this strain, PacBio RS II and Illumina HiSeq 2000 platforms were used for the whole genome sequencing. The genome size of strain FS91703 was 5,435,691 bp and GC content was 37.78%. A total of 4,951 coding genes were predicted; 99 potential virulence factors homologs were identified. Analysis of antibiotic resistance genes revealed that strain FS91703 harbored 10 antibiotic resistance genes in 6 categories and 2 multidrug-resistant efflux pump genes, including *adeG* and *farA*. Strain FS91703 was sensitive to β-lactam combination drugs, cephem, monobactam and carbapenems, intermediately resistant to phenicol, and resistant to penicillin, aminoglycosides, tetracycline, fluoroquinolones, and folate pathway inhibitors. Phylogenetic analysis revealed that strain FS91703 and *C. arthrosphaerae* CC-VM-7^T^ were on the same branch of the phylogenetic tree based on 16 S rRNA; the ANI value between them was 96.99%; and the DDH values were 80.2, 72.2 and 81.6% by three default calculation formulae. These results suggested that strain FS91703 was a species of *C. arthrosphaerae.* Pan-genome analysis showed FS91703 had 566 unique genes compared with 13 other *C. arthrosphaerae* strains, and had a distant phylogenetic relationship with the other *C. arthrosphaerae* strains of the same branch in phylogenetic tree based on orthologous genes. The results of this study suggest that strain FS91703 is a multidrug-resistant and highly virulent bacterium, that differs from other *C. arthrosphaerae* strains at the genomic level. The knowledge about the genomic characteristics and antimicrobial resistance of strain FS91703 provides valuable insights into this rare species, as well as guidance for the treatment of the disease caused by FS91703 in *Rana nigromaculata*.

**Supplementary Information:**

The online version contains supplementary material available at 10.1186/s12866-024-03223-6.

## Background

The genus *Chryseobacterium* belongs to the family *Flavobacteriaceae* [1], consisting of more than 100 species. These microorganisms are yellow-pigmented, non-spore-forming, non-motile, oxidase-positive, gram-negative, rod-shaped, and catalase-positive bacteria. They are abundant in water, soil, animals, and, plants. Among them, *C. meningoseptium*, *C. indogenes*, and *C. gellum* are considered as the most frequently isolated species from medical specimens [2]. The genus *Chryseobacterium* is an opportunistic pathogen that can cause meningitis, cellulitis, sepsis, and lower respiratory tract infections. The host range is very broad and includes humans and domestic animals [3, 4] as well as aquatic animals such as rainbow trout [5], snake eagles [6], sturgeon [7], and large pond turtles [2]. *Chryseobacteriums spp*. are resistant to cephalosporins, carbapenems, aminoglycosides, and polymyxin. Infections caused by these microorganisms pose a serious threat.

*C. arthrophaerae* is a rare species of the genus *Chryseobacterium*, originally identified in 2010 from the feces of *Arthrophaerae magna* (pill millipedes) [8]. The ED882-96 strain of *C. arthrosphaerae* was identified in 2019 from the blood of a clinical patient with liver cirrhosis, and showed resistance to all antibiotics tested; 83 virulence factor homologues were identified when compared with the virulence factor database (VFDB) [9]. This was the first study involving virulence factor in *C. arthrophaerae*. Im et al. found a clinical patient with meningitis caused by *C. artherosphaerae* in 2020 [10]. The compound sulfamethoxazole was then proposed as a treatment for this disease and the toxicity of this species to humans was confirmed [10].

Our previous study first reported *C. arthrosphaerae* isolates were obtained from ascites fluid of *Rana nigromaculata* [11]. And *C. arthrosphaerae* strain FS91703 is pathogenic to *Rana nigromaculata* [11]. There are no research reports on the isolation of *C. arthrosphaerae* from other frogs, and people have little understanding of the genetic information of this strain. In this study, the entire genome was sequenced and analyzed to determine the genomic characteristics and antimicrobial susceptibility of this strain. The results provide valuable insights into this rare species, as well as guidance for the treatment of the disease caused by *C. arthrosphaerae* FS91703 in *Rana nigromaculata*.

## Results

### Characterization of the genome of strain FS91703

The entire genome of strain FS91703 was sequenced and assembled, resulting in a final contig with 5,435,691 bp total length. The GC content was 37.78%, with 4,951 coding sequences. The coding genes was 4,754,943 bp length, with a 960 bp average length (87.48% of the total genome length). The interspersed repeats were 51 copies long, 3,208 bp (0.06% of the total genome length). Tandem repeats were 488 copies, 26,803 bp in length (0.49% of the total genome length). Strain FS91703 contained 94 tRNA genes, 18 rRNA genes (including 6 each of 23 S rRNA, 16 S rRNA, and 5 S rRNA), and 21 sRNA genes, with prophage 9, and the total length was 196 866 bp. The genome sequence and its annotation information were submitted to the NCBI database by accession number CP119767.

**Table 1:** Overview of genome function analysis of strain FS91703

Database	Gene number
NR	4 847
Swiss-Prot	2 388
KEGG	1 608
COG	3 041
GO	3 674
PHI	715
Pfam	14
VFDB	571
CARD	10
Secretory protein	658
T3SS	493
CAZY	145

### Genome functional analysis results

1,608, 3,674, 2,388, 3,041, and 4,847 genes were annotated in the KEGG, GO, Swiss-Prot, COG, and NR databases, respectively (Table 1). The minimum number of annotated genes was 10.

#### NR of strain FS91703

**Fig. 1:**
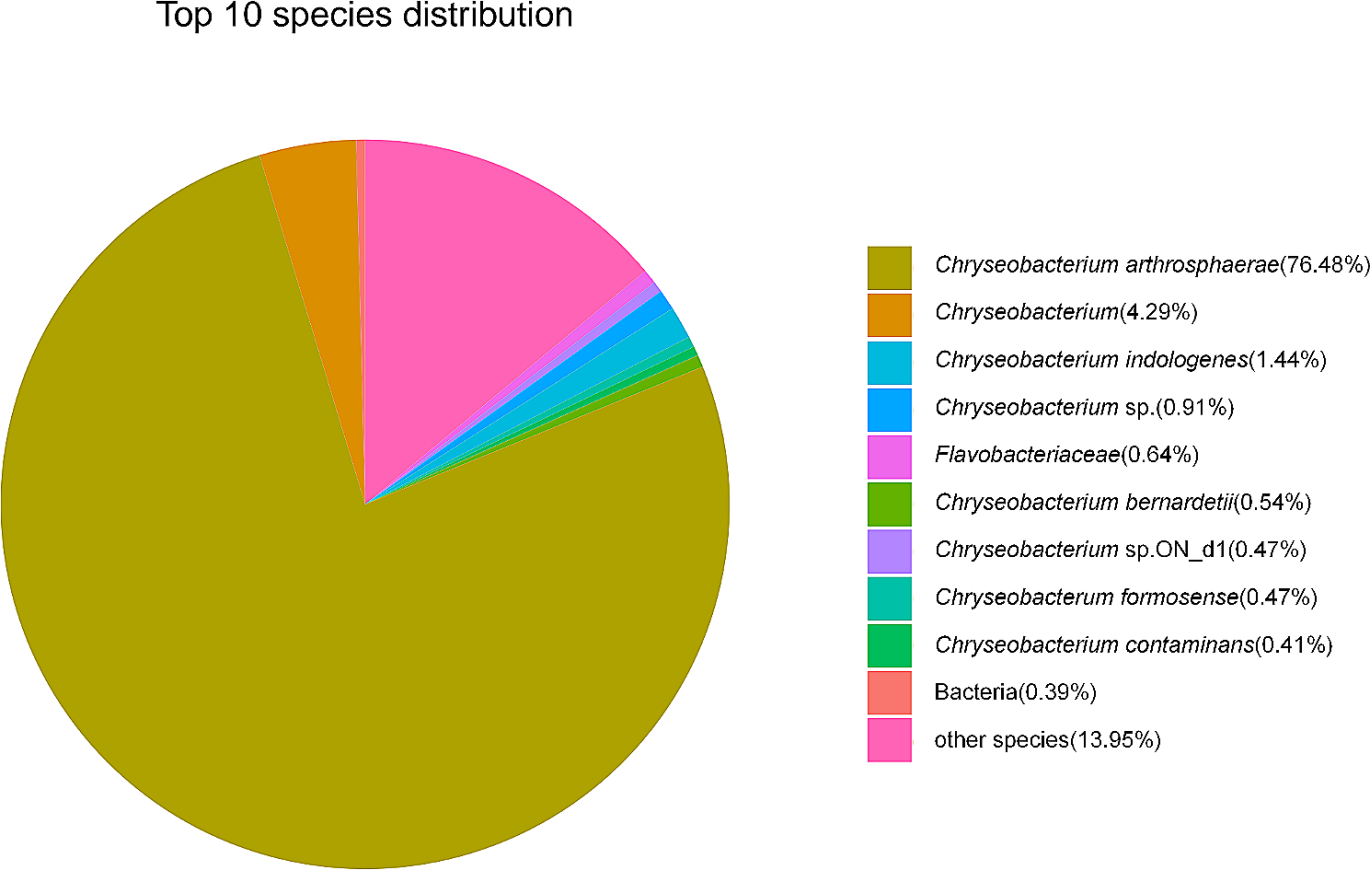
NR analysis result of strain FS91703. Different colored sectors indicate the percentage of each species

The gene sequence of strain FS91703 was converted to amino acid sequence and compared with the NR database. 4,847 genes were annotated in the NR database, of which the species of *C. artherosphaerae* was the mostly frequently annotated, accounting for 76.48% (Fig. 1).

#### GO of strain FS91703

**Fig. 2:**
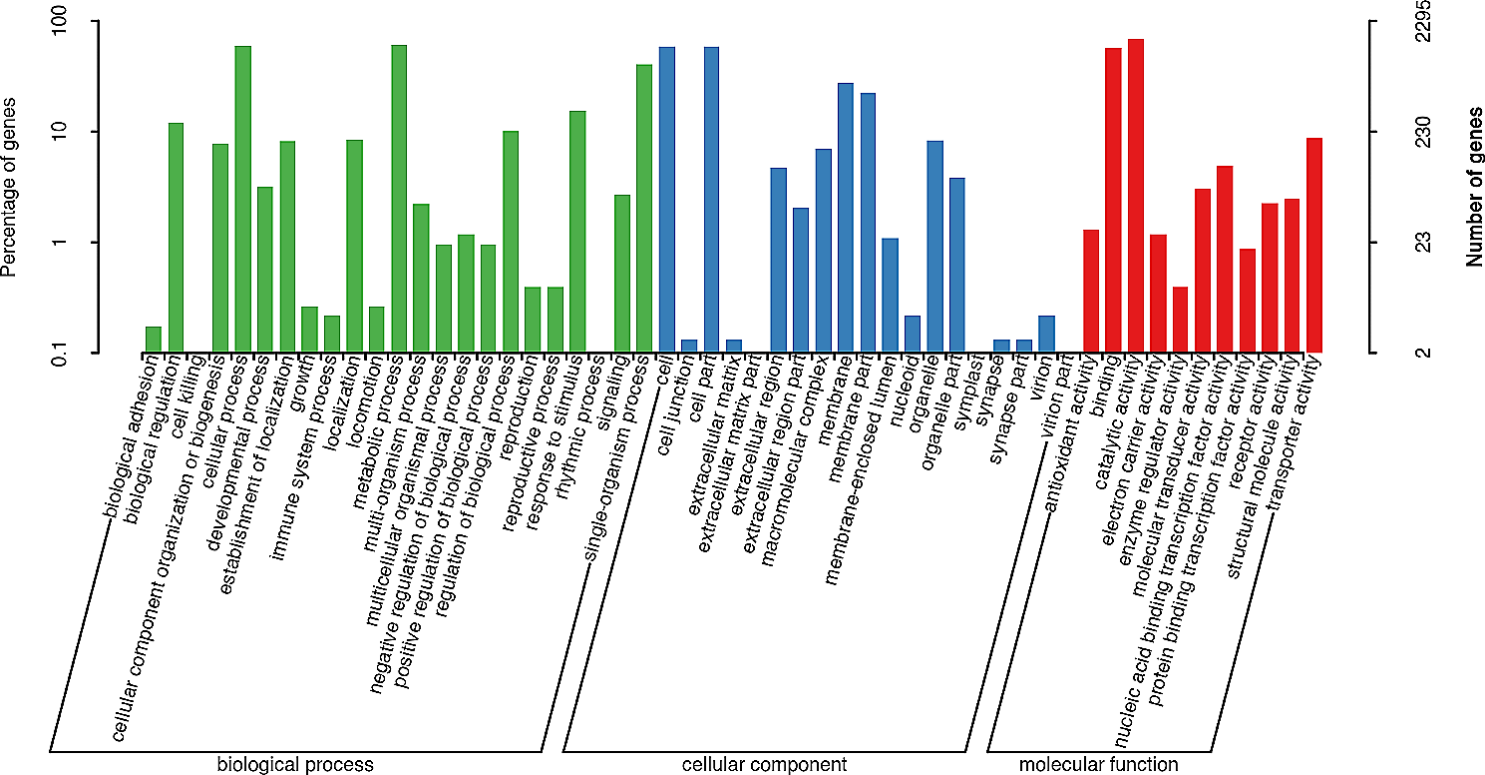
GO analysis for the result of strain FS91703

3,674 genes was annotated in the GO database. Cellular and metabolic processes in biological processes were two pathways with the highest gene enrichment of 1,376 and 1,393 genes, respectively. Cells and cell parts in cellular organizationhad the highest gene enrichment with 1,352 and 1,344 genes, respectively. Binding and catalytic activity were two pathways of molecular function with the greatest number of genesof 1,317 and 1,603 genes, respectively (Fig. 2).

**Fig. 3:**
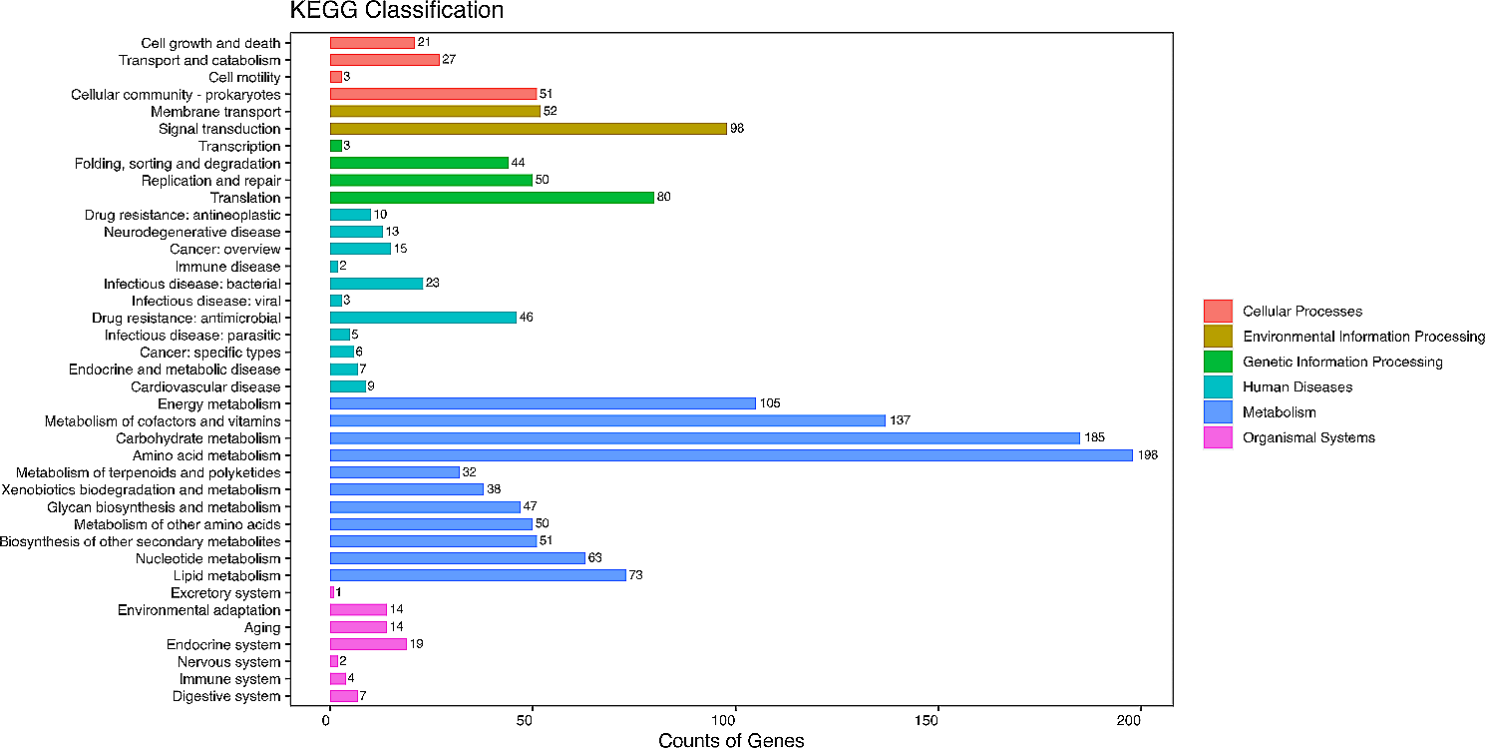
KEGG analysis for the result of strain FS91703

A total of 1,608 orthologous protein-coding genes were matched to 39 KEGG metabolic pathways. The pathways with the greatest number of genes were amino acid metabolism, carbohydrate metabolism, metabolism of cofactors and vitamin, which are necessary to maintain bacterial metabolism (Fig. 3).

**Fig. 4:**
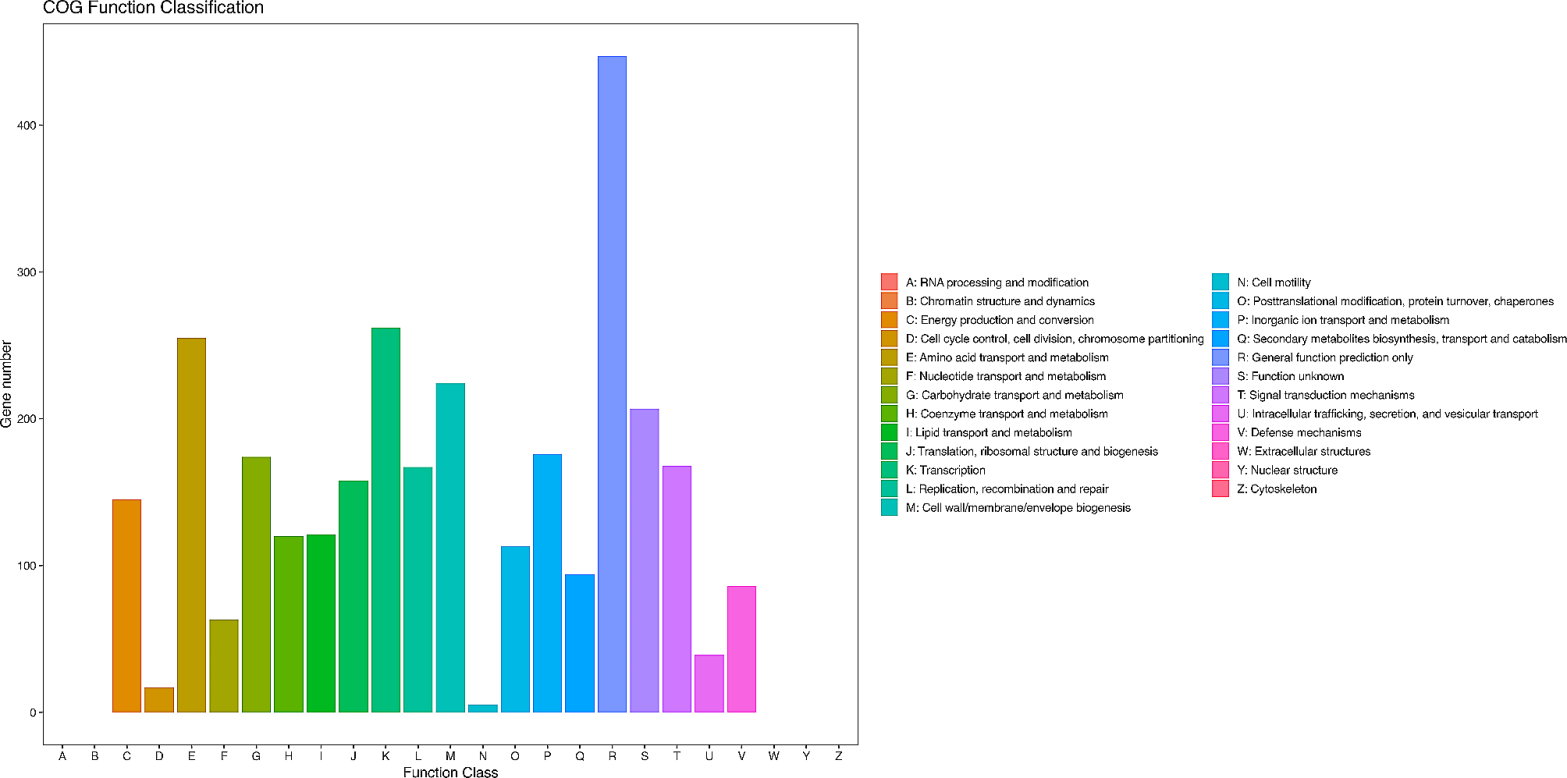
COG analysis for the result of strain FS91703

There were 3,041 genes annotated in the COG analysis. These genes were classified into 20 categories, C-V, according to the function. The annotated functions mainly covered the pathways of biosynthesis of cell wall, membrane, and envelope, metabolism, amino acid transport, transcription (Fig. 4). The results were similar to those of the KEGG metabolic pathway analysis. Many genes were found to be involved in metabolic processes that sustain basic bacterial life.

#### PHI of FS91703 strain

**Fig. 5:**
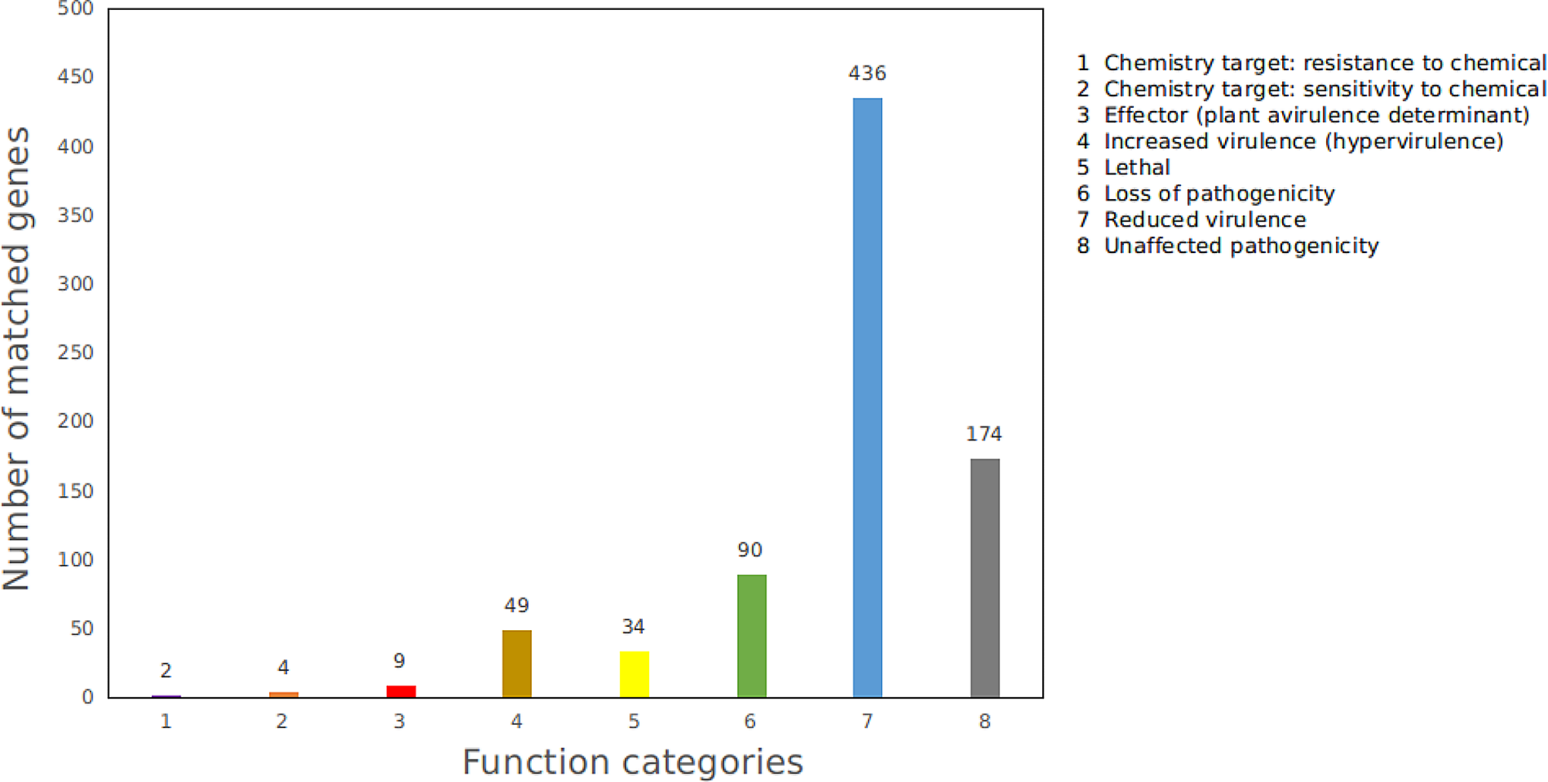
Distribution map of types of pathogen-host interaction genes of strain FS91703

The analysis of pathogen-host interaction-related genes showed that 798 genes annotated in the PHI database were classified into eight categories, with ‘reduced virulence’ (436 genes) being the largest category, followed by ‘unaffected pathogenicity’ (174 genes), ‘loss of pathogenicity’ (90 genes), ‘lethal’ (34 genes), ‘increased virulence (hypervirulence)’ (49 genes), ‘effector (plant avirulence determinant)’ ( 9 genes), ‘chemistry target: sensitivity to chemical’ (4 genes ), and ‘chemistry target: resistance to chemical’ ( 2 genes) (Fig. 5) In the process of infecting the host, pathogenic strain secretes a series of effectors, which plays an essential role in the interaction between the host and the pathogenic strain. The ability of effectors to effectively control the host is the key to the successful colonizationof pathogenic strains. Eight genes encoding effectors were annotated in the PHI database, including *clpV5* (3 genes), *lpdA* (2 genes), *mgtC* (2 genes) and *ipx10* (1 gene).

#### VFDB and CARD of FS91703 strain

Virulence factors are grouped into 14 categories according to their functions: regulation, antimicrobial activity/competitive advantage, post-translational modifications, stress survival, nutritional/metabolic factors, biofilms, immune modulation, exoenzymes, exotoxins, motility, effector delivery system, invasion, adhesion, and others. In this study, a total of 571 putative virulence genes were determined in strain FS91703 by DIAMOND analysis against the Virulence Factor Database (VFDB). Positive results were accepted with at least 40% identity, and e-value less than 1e-10. 99 potential virulence factors homologs were identified. The virulence factors with the highest number of annotated genes were immune modulators, stress survival factors, and nutritional/metabolic factors, with 39, 23, and 12 genes, respectively. Immune modulatory factors mainly included capsular, LPS O-antigen, LPS (lipopolysaccharide), and LOS (lipopolysaccharide). Among these factors, capsules had the highest number of encoding genes, with 9 such as *capK/L/5H*, *ndk*, *upps*, and *fnlA*. Stress resistance factors mainly included catalase, urease, ClpC, and MsrAB. There were three types of the effector secretory system: types III, IV, and VI. Of these, the most common virulence factors associated with the type III secretory system were T3SS, Psyringae TTSS effectors, and Mxi-Spa TTSS effectors regulated by MxiE. In the nutrient metabolism system, many factors related to iron uptake were found, including heme biosynthesis, aerobactin siderophores, and desferrioxamine. Four bacterial toxins were predicted: colibactin, β-hemolysin, the phytotoxin coronatine, and phosphatidylinositol-specific phospholipase C (PI-PLC). In addition, two drug efflux pump systems were annotated, namely AdeFGH and FarAB.

A total of 10 antibiotic resistance genes of strain FS91703 were annotated in CARD database. They are *sul2* (2 genes), *IND-14* (1 gene), *catB2* (1 gene), *catB6* (1 gene), *catB8* (1 gene), *tetX* (2 genes), *dfrE* (1 gene), and *streptomycetes* (1 gene), which are associated with the resistance of folate pathway inhibitors (*sul2/dfrE*), penicillin (*IND-14*), cephem (*IND-14*), carbapenems (*IND*-*14*), phenol (*catB2*, *catB6*, *catB8*), tetracycline (*tetX*), and ercomycin (*streptomyces*).

#### Genome-wide map of strain FS91703

**Fig. 6:**
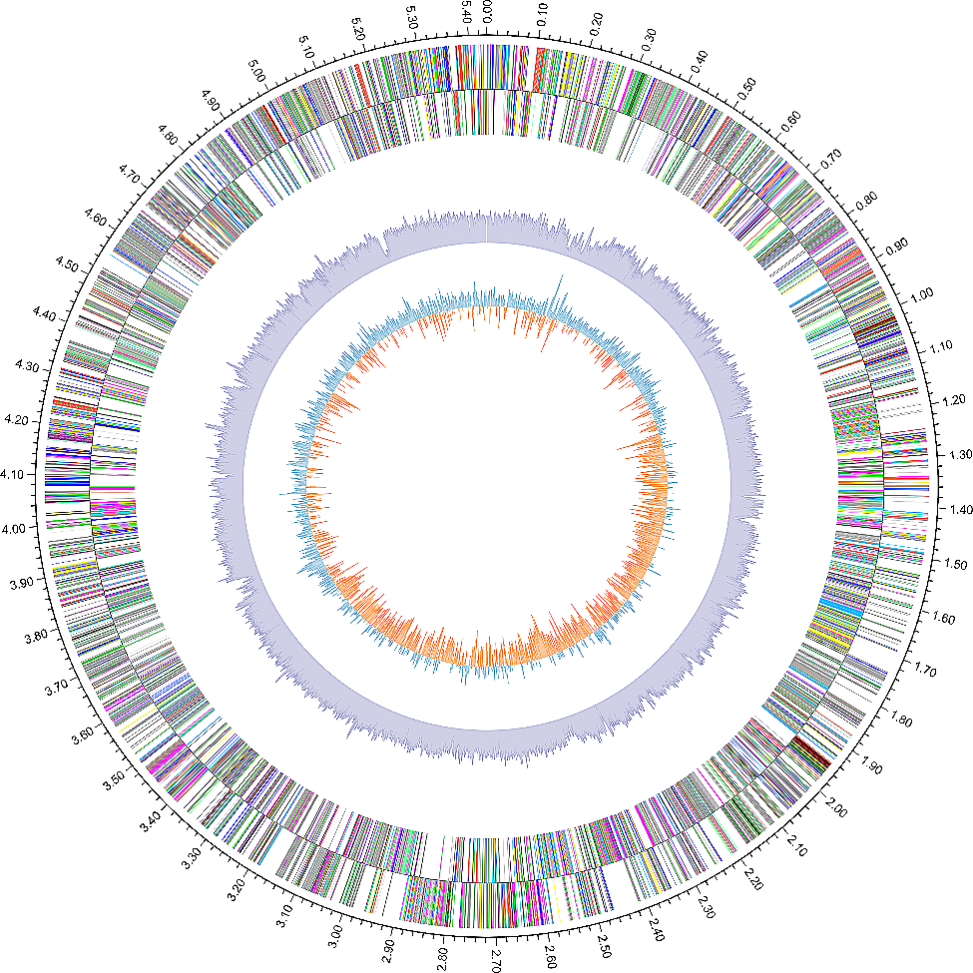
Genome-wide map of strain FS91703. Coding region prediction with color-coded by role category was indicated by circles. COG annotation, COG annotation for lagging strand, non-coding RNA (green, sRNA; blue, tRNA; red, rRNA), GC content, and GC skew for leading strand are listed from outside to inside

Based on basic genome sequence information, gene prediction results, non-coding RNA prediction results, and bioinformatics analysis results, we drew a whole genome map of this strain (Fig. 6).

#### Antimicrobial resistance of strain FS91703

The antimicrobial susceptibilities of strain FS91703 and MICs are shown in Table 2. Strain FS91703 was susceptible to β-lactam combination agents, cephems, monobactams, and carbapenems, and was intermediately resisitant to phenicol. It was also resistant to all other antimicrobial agents tested, including folate pathway inhibitors, fluoroquinolones, tetracyclines, aminoglycosides, and penicillin.

**Table 2:** Concentration of minimum growth inhibitory and antimicrobial susceptibility of strain FS91703

Antibiotics Group	Antibiotics	MIC	Interpretation
Penicillin	Piperacillin	≥ 128	R
β-lactam Combination Agents	Piperacillin-tazobactam	≤ 16/4	S
Cephems	Ceftazidime	≤ 8	S
	Cefepime	≤ 8	S
Monobactams	Aztreonam	≤ 8	S
Carbapenems	Imipenem	≤ 4	S
	Meropenem	≤ 4	S
Aminoglycosides	Gentamicin	≥ 16	R
	Tobramycin	≥ 16	R
	Amikacin	≥ 64	R
Tetracyclines	Tetracycline	≥ 16	R
	Minocycline	≥ 16	R
Fluoroquinolones	Cipofloxacin	≥ 4	R
	Levofloxacin	≥ 8	R
	Lomefloxactn	≥ 8	R
Inhibitors of folate pathway	Trimethoprim-sulfmethoxazole	≥ 4/76	R
Phenicol	Chloramphenicol	16	I

Note S-Sensitive, I-Intermediary, R-Resistant

### Phylogenetic relationships with 16 S rRNA

**Fig. 7:**
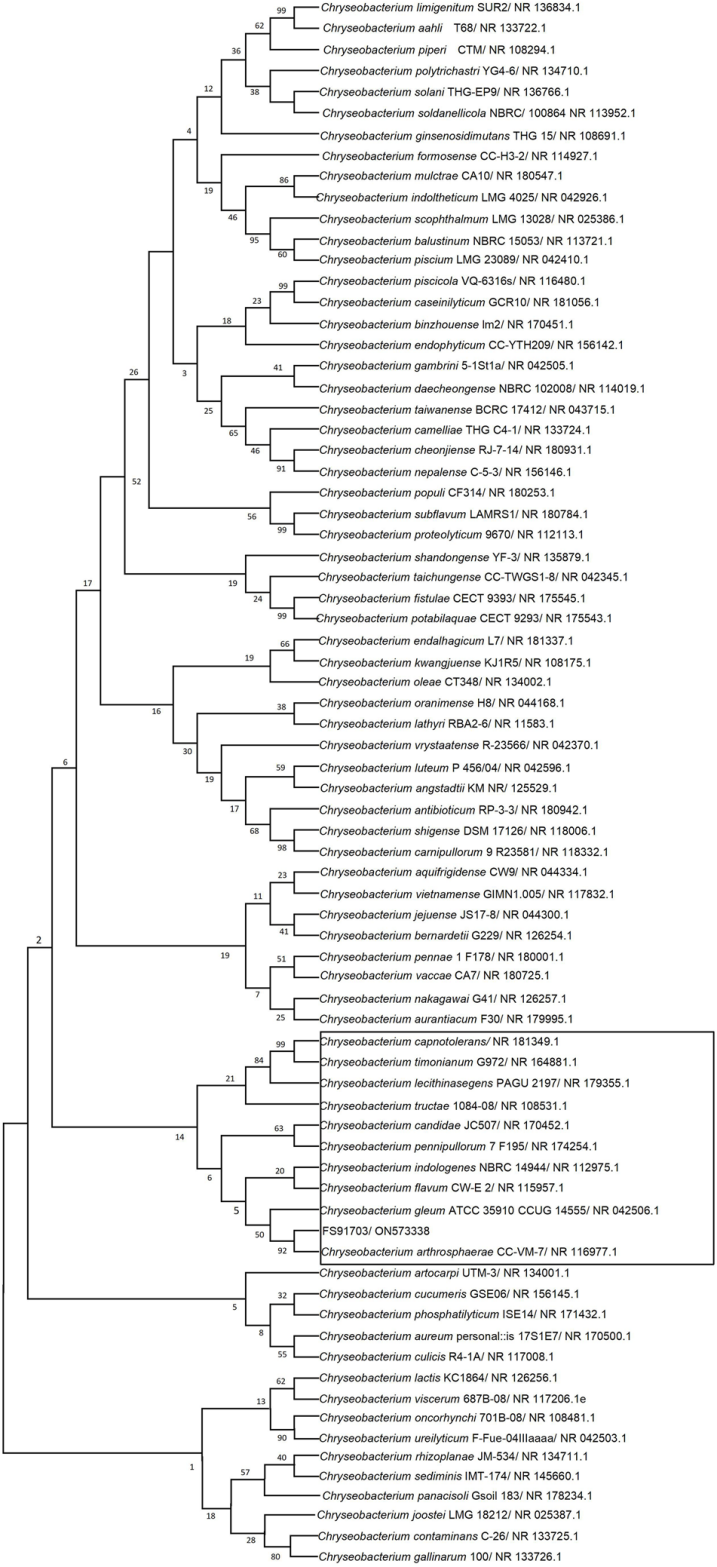
Phylogenetic tree with 16 S rRNA gene sequences of strain FS91703 and 74 type strains in the genus *Chryseobacterium*. The phylogenetic tree was constructed by the neighbour-joining method in MEGA 7. The numbers at the nodes of different branches indicate the bootstrap values, which are displayed in the form of percentage (%). Strains in the black rectangle are those for the analyses of ANI and in silico DDH

The 16 S rRNA of strain FS91703 contained 1406 base pairs and was submitted to GenBank (accession number: ON573338). The sequence was compared with those of other type strains registered in GenBank, and a phylogenetic tree was constructed by selecting species with high similarity (Fig. 7). Strain FS91703 shared the same branch as *C. arthrosphaerae* CC-VM-7^T^ strain, and the 16 S rRNA sequence of strain FS91703 was 99.08% identical to that of *C. arthrosphaerae* CC-VM-7^T^. These results suggest that strain FS91703 is one of the *C. arthrosphaerae* strains.

### Similarity of whole genomes

**Fig. 8:**
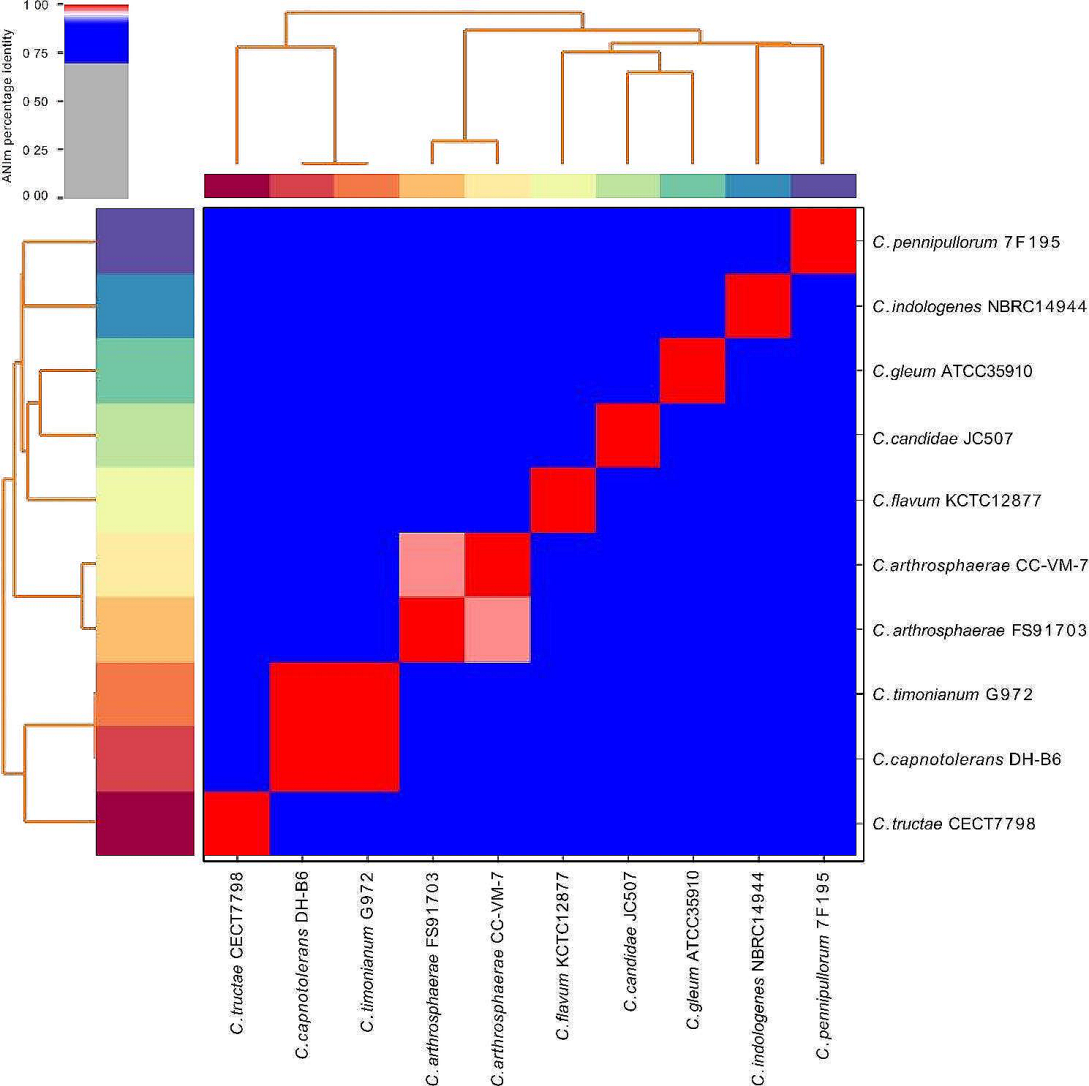
Heat map of the average nucleotide sequence identity (ANI) between strain FS91703 and 9 phylogentically close *Chryseobacterium* species

Strain FS91703 and 9 other phylogenetically close *Chryseobacterium* species with available whole genomes sequences (shown in the black rectangle of Fig. 6) were analyzed by ANI and in silico DDH. The ANI value between FS91703 and *C. arthrosphaerae* CC-VM-7^T^was 96.99%, and the values between strain FS91703 and other strains of the genus *Chryseobacterium* were less than 86% (Fig. 8). The DDH analysis between FS91703 and *C. arthrosphaerae* CC-VM-7^T^ using all three default calculation formulae suggested DDH values of 80.2, 72.2 and 81.6%, and DDH values > = 70% with the probabilities of 91.23, 81.95 and 96.66%, correspondingly(Table 3). These results demonstrated that strain FS91703 was a species of *C. arthrosphaerae*.

**Table 3:** DDH values between FS91703 and 9 phylogenetically close *Chryseobacterium* species

Strain compared to strain FS91703	Formula 1 (%)		Formula 2 (%)		Formula 3 (%)	
	DDH	Prob. DDH > = 70%	DDH	Prob. DDH > = 70%	DDH	Prob. DDH > = 70%
*C.arthrosphaerae* CC-VM-7	80.2	91.23	72.2	81.95	81.6	96.66
*C.candidae* JC507	32.8	0.23	27	0.03	30.4	0
*C.capnotolerans* DH-B6	32.2	0.19	25.4	0.01	29.5	0
*C.flavum* KCTC12877	30.3	0.1	25.2	0.01	28.1	0
*C.gleum* ATCC35910.CCUG14555	34	0.33	26.6	0.02	31.2	0
*C.indologenes* NBRC14944	30.4	0.1	24.8	0.01	28	0
*C.pennipullorum* 7F195	31	0.13	24.7	0.01	28.5	0
*C.timonianum* G972	31.9	0.17	25.4	0.01	29.3	0
*C.tructae* CECT7798	30.3	0.1	25.2	0.01	28.1	0

**Fig. 9:**
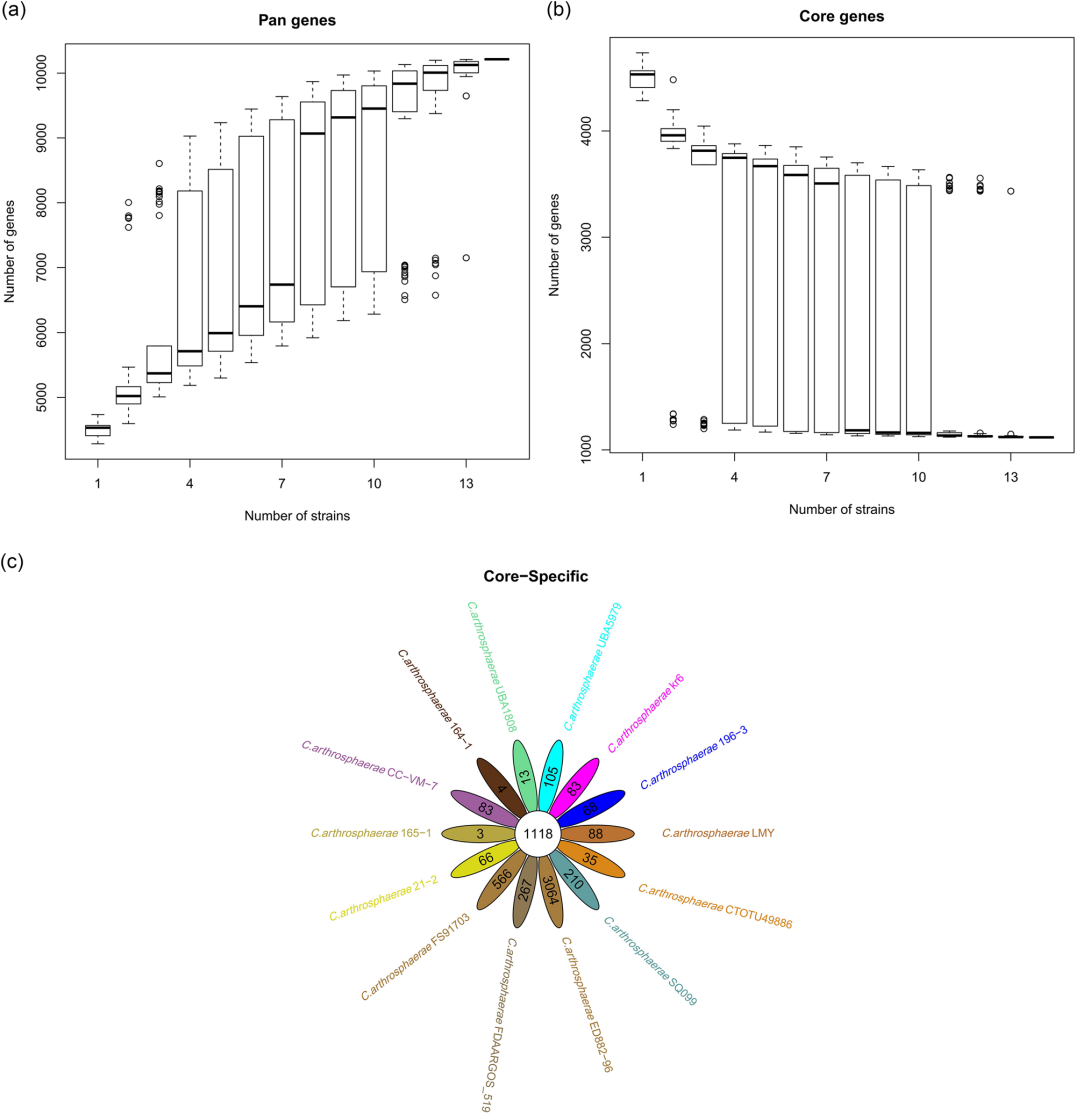
Analysis of pan-genome between FS91703 and other 13 *C*. *arthrosphaerae *strains. a. pan-genome characteristic curve, b. core-genome characteristic curve, c. flower plots showing the core gene number (in the center) and strain-specific gene number (in the petals)

**Fig. 10:**
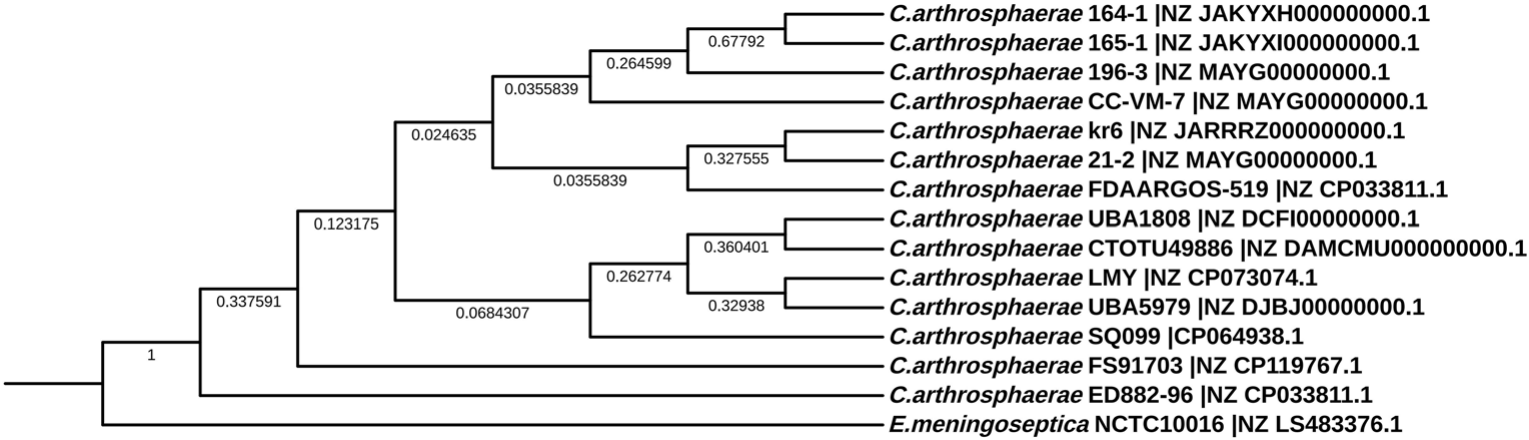
Phylogenetic tree of genome based on the single-copy orthologous genes of strain FS91703 and related strains. The phylogenetic tree was constructed by the maximum likelihood method in FastTree. The numbers at the nodes indicateof different branches indicated the bootstrap values

In order to display the pan-genome characteristics of 14 strains of *C. arthrosphaerae*, the pan-genome characteristic curves were plotted based on the clustering results (Fig. 9a and b). The results showed that as the number of strains increased, the pan-genome showed a significant increasing trend, indicating that *C. arthrosphaerae* had an open pan-genome. At the same time, as the numberof strains increased, the core-genome significantly decreased. To study the genomic differences of the species *C. arthrosphaerae*, we analyzed the distribution of core genes, non essential genes, and unique genes in each strain. The clustering results showed that the 14 strains shared a total of 1118 core genes. In addition, the number of unique genes present in each strain was 105(UBA5979), 13(UBA1808), 88(LMY), 3064(ED882-96), 4(164-1), 3(165-1), 267(FDAARGOS_519), 83(CC-VM-7), 35(CTOTU49886), 83(kr6), 66(21-2), 68(196-3), 210(SQ099), and 566(FS91703) (Fig. 9c). In order to elucidate the phylogenetic relationship of *C. arthrosphaerae* strains, a phylogenetic tree was constructed baesd on single-copy orthologous genes shared by 14 *C. arthrosphaerae* strains and *Elizabethkingia meningoseptica* strain NCTC10016 as the outgroup. 14 strains of *C. arthrosphaerae* generated two evolutionary directions. Strain ED882-96 formed an independent branch, while the other 13 strains formed another branch. There were also differences among the strains of the same branch, and FS91703 had a distant phylogenetic relationship with the other strains(Fig. 10).

## Discussion

As of December 18, 2023, the whole genome sequences of 14 *C. arthrosphaerae* strains have been uploaded to the NCBI database. However, studies on the biological characteristics of *C. arthrosphaerae* are still rare. Only the genomic characteristics of *C. arthrosphaerae* strain ED882-96, collected from the blood of a clinical patient, has been analyzed. Further studies on the host range, pathogenicity, virulence, and antibiotic screening of *C. arthrosphaerae* are urgently needed.

Virulence factors are important factors for bacterial virulence. 99 predicted virulence genes were annotated in strain FS91703 and 83 in strain ED882-96 by the VFDB database. Of these, 51 virulence genes encoding virulence factors such as LPS, capsule, effector delivery systems, colibactin, type IV pili, and polar flagella were identical in both strains. In addition, strain FS91703 had 48 unique virulence genes when compared with strain ED882-96. The difference in virulence genes between the two strains was shown in Table S1. Among the virulence genes in strain FS91703, 14 genes were involved in virulence factors of stress survival, including *katA*(Catalase), *katG*(Catalase-peroxidase), *ureG*/*B*/*A*(Urease), *sodB*(SodB), *mprA*(MprA/B), *msrA*/*BpilB*(MsrAB), *clpC*(ClpC) and *clpP*(ClpP). MsrAB catalyzes the reduction of protein-bound and free methionine sulfoxide (MetSO) to Met, as well as reparing oxidized proteins. Catalase, catalase-peroxidase, and SodB are important for intracellular survival and transmission, and this type of virulence factor makes microbes more resistant to oxidative stress [12–14]. *katG*, *clpP* and *msrA*/*BpilB* were unique virulence genes of strain FS91703 compared with strain ED882-96. The results indicate that there may be differences in virulence between strain FS91703 and strain ED882-96. According to our existing research, strain FS91703 could cause ascites disease in black frogs. Further research on pathogenicity of this strain will be investigated in our future studies.

The ED882-96 and FS91703 strains have different antibiotic resistance genes(Table S2) and different antibiotic susceptibilities. The ED882-96 strain is resistant to penicillin, aminoglycosides, tetracycline, fluoroquinolones, and folate pathway inhibitors [9], whereas the FS91703 strain is susceptible to β-lactam/β-lactamase inhibitor combinations, cephalosporins, monocyclic lactams, and carbapenem β-lactams. Im et al. also found that *C. artherosphaerae* strains with human meningitis were sensitive to ciprofloxacin (fluoroquinolone), minocycline (tetracycline), tetracycline, and compound sulfamethoxazole (folate pathway inhibitor), and cured their cases with compound sulfamethoxazole [10]. This indicates that there are differences in antibiotic resistance among the same species due to regional and host differences. In practice, antibiotic selection should be reasonably guided by antibiotic susceptibility results. The FS91703 strain also harbors genes such as *adeG* and *farA*, which encode multiple drug efflux pumps, AdeFGH and FarAB, respectively. Many studies have shown that drug efflux pumps can prevent drug accumulation in bacteria, creating internal bacterial resistance to toxic compounds such as antibiotics, disinfectants, detergents, and dyes. Drug efflux pump is one of the main mechanisms of multidrug resistance in bacteria: the AdeFGH efflux pump can efflux almost all antibiotics and lipids [15, 16]; the FarAB efflux pump can efflux long-chain fatty acids and some fat-soluble molecules [17]. No fluoroquinolone resistance genes were annotated in this study. However, strain FS91703 was resistant to fluoroquinolones such as cipofloxacin, levofloxacin, and lomefloxactn. The results suggest that the drug efflux pump may play an important role in the resistance of strain FS91703 to fluoroquinolones.

16 S rRNA sequence analysis, combined with ANI and in silico DDH analyses, confirmed that strain FS91703 was a species of *C. arthrosphaerae*. Pan-genome analysis showed FS91703 had 566 unique genes compared with 13 *C. arthrosphaerae* strains, and had a distant phylogenetic relationship with the other *C. arthrosphaerae* strains of the same branch in phylogenetic tree based on orthologous genes. The results indicate there are differences in genomic information between FS91703 and other *C. arthrosphaerae* strains. The differences in genomes may be related to the complex living environment of strains. Here, we obtained whole genome information on *C. arthrosphaerae* FS91703 strain from *Rana nigromaculata* for the first time. Whole genome analysis revealed that this strain is highly virulent and multidrug-resistant, with differences in virulence factors, drug resistance, and genomic information compared with other strains of *C. arthrosphaerae*. This study adds new information to the genome database of *C. arthrosphaerae*, enriches the host type of this species, and provides a reference for subsequent studies.

## Methods

### Strain FS91703

The recently deceased black-spotted frogs with ascites fluid were collected from a farm in Zhejiang Province of China. The ascites fluid was patterned on LB plates under sterile conditions, and then placed in a incubator at 37℃ for 48 h. A yellow colony of bacteria was isolated, named strain FS91703 and maintained at -80 °C in glycerol stock.

### Whole genome sequencing and assembly

The genome sequencing of strain FS91703 was performed by the the Illumina HiSeq 2000 platform (Illumina, San Diego, CA, USA) and PacBio RS II platform (Pacific Biosciences, Menlo Park, CA, USA). The sequence data (Subreads) were determined using the Illumina HiSeq 2000 platform (Illumina, San Diego, CA, USA). Subreads were self-corrected and assembled into genomes using Falcon [18]. Consistency sequences (Consumus) were first obtained based on the Overlap-Layout-Consense algorithm; Genomic Consumes was used and subreads were corrected again based on the arrow algorithm. Then, using the Corrected Subreads corrected with spai (single pass read accuracy improver) as auxiliary data, the assembled consensus sequences (Corrected Consumes) were cycled [19], and finally the cycled bacterial genome (Genome) was obtained.

### Bioinformatics Analysis

Genomic component predictions included coding gene, non-coding RNA, repeat sequence, and prophage predictions. Prokaryotic Dynamic Programming Genetic Algorithm (Prodigalv, 2.6.3), RepeatMask (v4.0.7) [20], PhiSpy (v2.3) [21] software were used to predict coding genes, repeat sequences, and prophages, respectively. Genomic components of rRNA, sRNA, and TRNA were predicted using RNAmmer (v1.2), Rfam (v10.0), and tRNAscan-SE (v1.3.1) software, respectively [22–24].

### Genome annotation and functional analysis

#### Common function database annotation

Functional genomic analysis was performed by on-line software, such as Cluster of orthologous groups of proteins (COG, https://www.ncbi.nlm.nih.gov/COG/), Non-Redundant Protein Database (NR, https://www.ncbi.nlm.nih.gov/), Swiss-Prot (http://www.uniprot.org/), Kyoto Encyclopedia of Genes and Genomes (KEGG, http://www.genome.jp/kegg/pathway.html) [25], Carbohydrate-Active enZYmes Database (CAZy, http://www.cazy.org) [26], Gene Ontology (GO), Pfam(http://pfam.xfam.org/)database, and evolutionary genealogy of genes (eggNOG, http://eggnog.embl.de/). The annotation of the KEGG, eggNOG, Swissprot, GO, COG, and NR databases was performed for the comparison using the DIAMOND [27] software, and the proteins with the highest sequence similarity with annotations e < 1e-5 were selected to gain the information of functional annotation. HMMER [28] software and protein family models were used to compare the annotations in the Pfam and CAZy. The annotations were compared and the protein families with the highest scores were screened.

#### Other function database annotation

The Pathogen host interactions (PHI, http://www.phi-base.org/) database [29] was used to analyze pathogen-host interactions. Pathogenicity factors were predicted using the virulence factors of pathogenic bacteria (VFDB, http://www.mgc.ac.cn/VFs/main.htm) [30]. The comprehensive antibiotic research database (CARD, https://card.mcmaster.ca/) was used for genes related to antibiotic resistance [31]. DIAMOND and BLAST software was used to compare predicted coding sequences, taking annotations with e < 1e-10 [27, 32].

#### Genome-wide map of strain FS91703

The circos (v0.69) software was used for the circular genome to create a graphical map [33].

### Antimicrobial susceptibility

The microdilution method was used for the determination of minimum inhibitory concentration (MIC). The criteria for “other non-*Enterobacteriaceae*” based on Guidelines of Clinical and Laboratory Stanards Institute (CLSI) were used for the interpretation of antibiotic susceptibility [34].

### Phylogenetic Tree based on 16 S rRNA gene sequences

The sequence of 16 S rRNA gene of strain FS91703 was obtained from the whole genome sequencing results. BLAST comparison was performed in NCBI, and homologous sequences of type strains with high similarity were selected. The Neighbor-Joining method [35] in MEGA 7.0 software was used for constructing the phylogenetic tree [36].

### Analysis of genome similarity

Whole-genome similarity was analyzed using average nuleotide identity (ANI), in silico DNA-DNA hybridization (DDH) and pan-genome analysis. ANI is one of the key indicators of reflect genetic distance. In general, 95% of the ANI value is considered the criterion for separating the same species [37]. Pyani (v0.2.12) was used to calculate ANI values [38], while in silico DDH values were evaluated using the online Genome-to-Genome Distance Calculator (GGDC 2.0 tool, http://ggdc.dsmz.de/distcalc2.php) [39]. The cutoff value of 70% was recommended as criterion for species delimitation [39].

Cluster Analysis of genomic protein sequences in strain FS91703 and other 13 strains of *C. arthrosphaerae* with available whole genomes sequences in NCBI was performed using CD-HIT (v4.6.6). The information of core genes, non essential genes, and specific genes for each strain was collected from the clustering results. To gain a deeper understanding of the evolutionary relationships of the *C. arthrosphaerae* strains, *Elizabethkingia meningoseptica* strain NCTC10016 was selected as the outgroup, gene families were constructed using OrthoFinder (v 2.5.4). DIAMOND was used for multiple sequence alignment, and the phylogenetic tree of orthologous genes was constructed by the maximum likelihood method in FastTree.

### Electronic supplementary material

Below is the link to the electronic supplementary material.

Supplementary Material 1: Table S1: Virulence genes predicted using Virulence Factors Database (VFDB) of strain FS91703

Supplementary Material 2: Table S2: Differences in genes associated with antibiotic resisitance using Comprehensive Antibiotic Resisitance Database (CARD) between FS91703 and ED882-96 strains

## Data Availability

The complete data set was submitted to the National Biotechnology Information Center (NCBI) database with the accession number CP119767. Data has been uploaded to the website https://www.ncbi.nlm.nih.gov/bioproject/PRJNA943546.
