# Supplementary material for: Whole-genome sequencing and analysis of Chryseobacterium arthrosphaerae from Rana nigromaculata
Source: BMC Microbiol. 2024 Mar 8;24:80. doi: 10.1186/s12866-024-03223-6 (PMC10921761; doi:10.1186/s12866-024-03223-6)
Supplement: Supplementary file 1 — Supplementary Material 1: Table S1: Virulence genes predicted using Virulence Factors Database (VFDB) of strain FS91703 [file 12866_2024_3223_MOESM1_ESM.doc]

Table S1. Virulence genes predicted using Virulence Factors Database (VFDB) of strain FS91703

| No. | Identity | E value | VF type ID | VF Description | VF Name | VF species | strain ED882-96 |
| --- | --- | --- | --- | --- | --- | --- | --- |
| 1 | 42.10 | 3.90E-151 | iutA | IutA (CVF457) | Aerobactin siderophore | *Escherichia coli* APEC O1 | - |
| 2 | 44.63 | 9.40E-77 | wbpE | probable aminotransferase WbpE (CVF520) | LPS O-antigen | *Pseudomonas aeruginosa* PAO1 | + |
| 3 | 51.50 | 1.40E-95 | galE | UDP-glucose 4-epimerase (CVF494) | LOS | *Haemophilus somnus* 2336 | - |
| 4 | 42.86 | 1.10E-45 | PSPTO_4713 | alanyl tRNA synthetase-related protein (CVF545) | Phytotoxin coronatine | *Pseudomonas syringae* pv. *tomato* str. DC3000 | - |
| 5 | 42.11 | 1.00E-24 | mlr6326 | putative DNA invertase (SS026) | T3SS | *Mesorhizobium loti* MAFF303099 | - |
| 6 | 46.62 | 4.80E-26 | ndk | Probable nucleoside diphosphate kinase (NDK) (CVF660) | Nucleoside diphosphate kinase | *Mycobacterium abscessus* ATCC 19977 | +^*^ |
| 7 | 47.53 | 4.60E-39 | lirB | Dot/Icm type IV secretion system effector LirB (SS047) | Dot/Icm | *Legionella pneumophila* subsp. *pneumophila* str. *Philadelphia* 1 | + |
| 8 | 43.90 | 1.40E-83 | pilR | putative two-component system, response regulator (CVF518) | Type IV pili biosynthesis | *Pseudomonas fluorescens* SBW25 | + |
| 9 | 41.36 | 3.60E-136 | ctpV | Putative metal cation transporter P-type ATPase CtpV (CVF658) | Copper exporter | *Mycobacterium canettii* CIPT 140070010 | - |
| 10 | 40.30 | 1.30E-34 | hisH2 | glutamine amidotransferase (CVF520) | LPS O-antigen | *Pseudomonas aeruginosa* PAO1 | + |
| 11 | 45.82 | 1.30E-58 | hisF | imidazole glycerol phosphate synthase subunit HisF (VF0171) | LPS | *Legionella pneumophila* subsp. *pneumophil*a str. *Philadelphia* 1 | + |
| 12 | 45.56 | 1.40E-37 | leuD | 3-isopropylmalate dehydratase, small subunit (CVF309) | Leucine synthesis | *Mycobacterium* sp. JLS | - |
| 13 | 44.14 | 2.30E-103 | katA | catalase (CVF760) | Catalase | *Neisseria lactamica* 020-06 | + |
| 14 | 45.23 | 6.80E-118 | dfoJ | putative decarboxylase involved in desferrioxamine biosynthesis (IA033) | desferrioxamine | *Erwinia amylovora* CFBP1430 | + |
| 15 | 44.06 | 1.00E-106 | dfoA | L-lysine 6-monooxygenase involved in desferrioxamine biosynthesis (IA033) | desferrioxamine | *Erwinia amylovora* CFBP1430 | + |
| 16 | 45.80 | 9.30E-27 | msrA/BpilB | trifunctional thioredoxin/methionine sulfoxide reductase A/B protein (VF0456) | MsrAB | *Neisseria meningitidis* MC58 | - |
| 17 | 49.22 | 3.00E-64 | kdsA | 2-dehydro-3-deoxyphosphooctonate aldolase (CVF383) | LPS | *Brucella melitensis* bv. 1 str. 16M | +^*^ |
| 18 | 43.41 | 4.00E-68 | kpsF | arabinose-5-phosphate isomerase (CVF393) | Capsule biosynthesis and transport | *Campylobacter jejuni* RM1221 | +^*^ |
| 19 | 41.90 | 2.40E-59 | clbD | putative 3-hydroxyacyl-CoA dehydrogenase (TX033) | colibactin | *Escherichia coli* O18:K1:H7 str. IHE3034 | + |
| 20 | 68.37 | 7.00E-16 | evpJ | type VI secretion system protein EvpJ (SS189) | EVP | *Edwardsiella tarda* EIB202 | + |
| 21 | 54.23 | 6.20E-78 | IlpA | immunogenic lipoprotein A (VF0513) | IlpA | *Vibrio vulnificus* YJ016 | + |
| 22 | 74.37 | 5.20E-88 | ureG | urease/hydrogenase-associated predicted GTPase UreG (CVF221) | Urease | *Helicobacter hepaticus* ATCC 51449 | + |
| 23 | 41.46 | 7.20E-24 | ureE | urease accessory protein UreE (CVF221) | Urease | *Helicobacter pylori* Shi470 | + |
| 24 | 68.48 | 7.90E-232 | ureB | urease (CVF221) | Urease | *Helicobacter hepaticus* ATCC 51449 | + |
| 25 | 59.66 | 4.80E-38 | ureA | urease alpha subunit (CVF221) | Urease | *Helicobacter acinonychis* str. Sheeba | + |
| 26 | 69.00 | 1.40E-30 | ureA | urease alpha subunit(CVF221) | Urease | *Helicobacter acinonychis* str. Sheeba | + |
| 27 | 43.26 | 1.70E-22 | farA | putative efflux pump protein (CVF758) | FarAB | *Neisseria meningitidis* alpha14 | + |
| 28 | 55.53 | 5.30E-132 | hemL | glutamate-1-semialdehyde aminotransferase (CVF506) | Heme biosynthesis | *Haemophilus somnus* 129PT | + |
| 29 | 62.02 | 7.00E-197 | CT396 | molecular chaperone DnaK (AI392) | MOMP | *Chlamydia trachomatis* D/UW-3/CX | + |
| 30 | 51.09 | 1.90E-89 | hemB | Porphobilinogen synthase (CVF506) | Heme biosynthesis | *Haemophilus somnus* 2336 | + |
| 31 | 42.33 | 3.00E-29 | lpxA | UDP-N-acetylglucosamine acyltransferase (CVF494) | LOS | *Haemophilus ducreyi* 35000HP | - |
| 32 | 52.82 | 1.40E-18 | lpxD | lpxD (CVF774) | LPS | *Acinetobacter baumannii* 1656-2 | - |
| 33 | 52.22 | 8.50E-47 | rmlC | dTDP-6-deoxy-D-xylo-4-hexulose-3,5-epimerase (CVF282) | Capsular polysaccharide | *Vibrio vulnificus* YJ016 | - |
| 34 | 55.61 | 6.20E-116 | capL | hypothetical protein (CVF110) | Capsule | *Staphylococcus haemolyticus* JCSC1435 | + |
| 35 | 47.25 | 1.70E-37 | BCE_5393 | UDP-galactose phosphate transferase (CVF567) | Polysaccharide capsule | *Bacillus cereus* ATCC 10987 | + |
| 36 | 47.22 | 2.90E-95 | capK | hypothetical protein (CVF110) | Capsule | *Staphylococcus haemolyticus* JCSC1435 | + |
| 37 | 70.82 | 3.20E-157 | ABTJ_03752 | UDP-N-acetylglucosamine 2-epimerase (CVF775) | Capsule | *Acinetobacter baumannii* MDR-TJ | + |
| 38 | 68.44 | 2.60E-129 | fnlA | UDP-N-acetylglucosamine 4,6-dehydratase (CVF775) | Capsule | *Acinetobacter baumannii* D1279779 | + |
| 39 | 56.52 | 4.30E-81 | PSPA7_1979 | imidazole glycerol phosphate synthase subunit HisF (CVF520) | LPS O-antigen | *Pseudomonas aeruginosa* PA7 | + |
| 40 | 44.12 | 5.60E-47 | hisH2 | glutamine amidotransferase (CVF520) | LPS O-antigen | *Pseudomonas aeruginosa* PAO1 | + |
| 41 | 74.01 | 3.20E-173 | PSPA7_1978 | LPS biosynthesis protein WbpG (CVF520) | LPS O-antigen | *Pseudomonas aeruginosa* PA7 | - |
| 42 | 62.03 | 1.00E-42 | wbpD | probable acetyltransferase WbpD (CVF520) | LPS O-antigen | Pseudomonas aeruginosa PAO1 | + |
| 43 | 47.63 | 2.40E-80 | wbpE | probable aminotransferase WbpE (CVF520) | LPS O-antigen | *Pseudomonas aeruginosa* PAO1 | - |
| 44 | 44.36 | 2.60E-101 | ipaH | hypothetical protein (CVF465) | Mxi-Spa TTSS effectors controlled by MxiE | *Shigella flexneri* 2a str. 2457T | + |
| 45 | 57.04 | 3.20E-145 | PLES_19091 | UDP-glucose/GDP-mannose dehydrogenase-like protein (CVF520) | LPS O-antigen | *Pseudomonas aeruginosa* LESB58 | + |
| 46 | 40.88 | 1.60E-29 | aatC | ABC transporter ATP-binding protein (CVF737) | ABC transporter for dispersin | *Escherichia coli* O44:H18 042 | +^*^ |
| 47 | 54.13 | 1.30E-27 | panD | aspartate 1-decarboxylase (CVF305) | Pantothenate synthesis | *Mycobacterium abscessus* subsp. *bolletii* 50594 | +^*^ |
| 48 | 44.37 | 1.60E-67 | pdhB | pyruvate dehydrogenase E1 component, beta subunit (CVF588) | PDH-B | *Mycoplasma synoviae* 53 | +^*^ |
| 49 | 61.34 | 9.00E-148 | icl | isocitrate lyase (CVF302) | Isocitrate lyase | *Mycobacterium avium* 104 | + |
| 50 | 64.89 | 7.90E-280 | katG | catalase (CVF321) | Catalase-peroxidase | *Mycobacterium indicus pranii* MTCC 9506 | - |
| 51 | 57.94 | 4.30E-34 | mgtC | hypothetical protein (CVF005) | Mg2+ transport | *Salmonella enterica* subsp. *arizonae serovar* 62:z4,z23:-- str. RSK2980 | + |
| 52 | 48.81 | 5.30E-71 | MG_301 | glyceraldehyde-3-phosphate dehydrogenase (AI368) | GAPDH | *Mycoplasma genitalium* G37 | + |
| 53 | 65.54 | 3.50E-189 | htpB | Hsp60, 60K heat shock protein HtpB (CVF347) | Hsp60 | *Legionella pneumophila* str. Corby | + |
| 54 | 67.18 | 3.90E-161 | tuf | translation elongation factor Tu (CVF587) | EF-Tu | *Mycoplasma mycoides* subsp. *mycoides* SC str. PG1 | + |
| 55 | 58.67 | 2.80E-18 | acpXL | acyl carrier protein (CVF383) | LPS | *Brucella melitensis* bv. 1 str. 16M | + |
| 56 | 41.81 | 3.10E-59 | rmlD | dTDP-4-dehydrorhamnose reductase (CVF282) | Capsular polysaccharide | *Vibrio vulnificus* YJ016 | + |
| 57 | 43.10 | 4.10E-52 | uppS | undecaprenyl diphosphate synthase (CVF618) | Capsule | *Enterococcus faecium* Aus0004 | - |
| 58 | 40.50 | 1.90E-32 | BCE_5400 | capsular exopolysaccharide family protein (CVF567) | Polysaccharide capsule | *Bacillus cereus* ATCC 10987 | + |
| 59 | 40.08 | 5.90E-38 | SSU05_0574 | Glycosyltransferases involved in cell wall biogenesis (CVF186) | Capsule | *Streptococcus suis* 05ZYH33 | - |
| 60 | 45.31 | 3.90E-47 | cylG | 3-ketoacyl-ACP-reductase CylG (CVF171) | Beta-hemolysin/cytolysin | *Streptococcus agalactiae* 2603V/R | - |
| 61 | 54.36 | 4.50E-151 | katA | catalase (CVF760) | Catalase | *Neisseria lactamica* 020-06 | - |
| 62 | 45.85 | 1.50E-51 | qbsC | QbsC (IA009) | thioquinolobactin | *Pseudomonas fluorescens* ATCC 17400 | - |
| 63 | 61.93 | 3.60E-202 | pgi | Glucose-6-phosphate isomerase (CVF495) | Exopolysaccharide | *Haemophilus somnus* 2336 | +^*^ |
| 64 | 46.04 | 7.40E-65 | fleQ | transcriptional regulator FleQ (AI149) | polar flagella | *Legionella pneumophila* subsp. *pneumophil*a str. Philadelphia 1 | - |
| 65 | 45.51 | 2.90E-34 | msrA/BpilB | trifunctional thioredoxin/methionine sulfoxide reductase A/B protein (VF0456) | MsrAB | *Neisseria meningitidis* MC58 | - |
| 66 | 41.88 | 3.90E-52 | mprA | two component transcriptional regulator, winged helix family (CVF333) | MprA/B | *Mycobacterium gilvu*m PYR-GCK | - |
| 67 | 51.46 | 7.00E-60 | flmH | 3-oxoacyl-ACP reductase (VF0473) | Polar flagella | *Aeromonas hydrophila* ML09-119 | + |
| 68 | 47.49 | 6.60E-211 | clpC | endopeptidase Clp ATP-binding chain C (VF0072) | ClpC | *Listeria monocytogenes* EGD-e | + |
| 69 | 53.73 | 8.50E-150 | katA | catalase (CVF760) | Catalase | *Neisseria lactamica* 020-06 | - |
| 70 | 44.07 | 2.10E-108 | katA | catalase(CVF760) | Catalase | *Neisseria lactamica* FA 1090 | - |
| 71 | 54.24 | 2.30E-85 | piplc | 1-phosphatidylinositol phosphodiesterase precursor (CVF570) | Phosphatidylinositol-specific phospholipase C (PI-PLC) | *Bacillus cereus ATCC* 14579 | +^*^ |
| 72 | 44.90 | 3.10E-82 | hemE | uroporphyrinogen decarboxylase (CVF506) | Heme biosynthesis | *Haemophilus somnus* 2336 | - |
| 73 | 43.90 | 1.70E-23 | msrA/BpilB | trifunctional thioredoxin/methionine sulfoxide reductase A/B protein(VF0456) | MsrAB | *Neisseria meningitidis MC58* | - |
| 74 | 45.03 | 1.90E-47 | sodB | superoxide dismutase (VF0169) | SodB | *Legionella pneumophila* subsp. *pneumophila* str. Philadelphia 1 | - |
| 75 | 43.14 | 1.20E-41 | mig-5 | putative carbonic anhydrase (VF0396) | Mig-5 | *Salmonella enterica* subsp. *enterica serovar* Typhimurium str. LT2 | - |
| 76 | 40.98 | 7.90E-16 | cysE | serine acetyltransferase (CVF567) | Polysaccharide capsule | *Bacillus thuringiensis* serovar *konkukian* str. 97-27 | - |
| 77 | 61.99 | 4.40E-127 | M3Q_290 | hypothetical protein (CVF775) | Capsule | *Acinetobacter baumannii* TYTH-1 | - |
| 78 | 76.58 | 7.60E-11 | C8J_1080 | hypothetical protein (CVF396) | LOS | *Campylobacter jejuni* subsp. *jejuni* 81116 | - |
| 79 | 45.80 | 3.60E-29 | C8J_1079 | hypothetical protein (CVF396) | LOS | *Campylobacter jejuni* subsp. *jejuni* 81116 | - |
| 80 | 40.65 | 1.80E-25 | C8J_1079 | hypothetical protein(CVF396) | LOS | *Campylobacter jejuni* subsp. *jejuni* 81116 | - |
| 81 | 65.05 | 1.20E-108 | rmlA | Glucose-1-phosphate thymidylyltransferase (CVF282) | Capsular polysaccharide | *Vibrio fischeri* ES114 | - |
| 82 | 56.70 | 6.50E-115 | rmlB | dTDP-glucose 4,6-dehydratase (CVF282) | Capsular polysaccharide | *Vibrio fischeri* ES114 | - |
| 83 | 49.21 | 1.00E-122 | BJAB07104_00096 | putative UDP-glucose 6-dehydrogenase (CVF775) | Capsule | *Acinetobacter baumannii* BJAB07104 | - |
| 84 | 42.33 | 3.90E-41 | sodB | superoxide dismutase (VF0169) | SodB | *Legionella pneumophila* subsp. *pneumophila* str. *Philadelphia* 1 | - |
| 85 | 67.60 | 2.60E-163 | eno | enolase (CVF153) | Streptococcal enolase | *Streptococcus gordonii* str. Challis substr. CH1 | - |
| 86 | 46.81 | 4.00E-186 | clpC | endopeptidase Clp ATP-binding chain C (VF0072) | ClpC | *Listeria monocytogenes* EGD-e | - |
| 87 | 44.17 | 6.00E-20 | cap5H | capsular polysaccharide biosynthesis protein Cap5H (CVF110) | Capsule | *Staphylococcus aureus* subsp. *aureus* USA300_FPR3757 | - |
| 88 | 43.62 | 6.60E-32 | kdtB | lipopolysaccharide core biosynthesis protein (VF0056) | LPS | *Helicobacter pylori* 26695 | + |
| 89 | 47.55 | 1.10E-33 | mip | macrophage infectivity potentiator (CVF349) | Mip | *Legionella longbeachae* NSW150 | + |
| 90 | 40.22 | 1.70E-88 | pilR | putative two-component system, response regulator (CVF518) | Type IV pili biosynthesis | *Pseudomonas fluorescens* SBW25 | - |
| 91 | 44.04 | 4.20E-19 | bscD | putative outer membrane protein (SS193) | T6SS | *Burkholderia cenocepacia* J2315 | - |
| 92 | 41.77 | 2.30E-145 | zmp1 | endothelin-converting enzyme (CVF655) | Zn++ metallophrotease | *Mycobacterium smegmatis* JS623 | - |
| 93 | 41.77 | 1.20E-141 | zmp1 | endothelin-converting enzyme(CVF655) | Zn++ metallophrotease | *Mycobacterium gilvum Spyr1* | - |
| 94 | 40.73 | 3.00E-213 | adeG | RND cation/multidrug efflux pump (CVF773) | AdeFGH efflux pump/transport autoinducer | *Acinetobacter baumannii* D1279779 | +^*^ |
| 95 | 51.04 | 6.40E-21 | hopJ1 | type III effector HopJ1 (CVF534) | P. syringae TTSS effectors | *Pseudomonas syringae* pv. *syringae* B728a | - |
| 96 | 52.38 | 9.00E-54 | clpP | ATP-dependent Clp protease proteolytic subunit (VF0074) | ClpP | *Listeria monocytogenes* EGD-e | - |
| 97 | 52.52 | 6.00E-100 | msrA/BpilB | trifunctional thioredoxin/methionine sulfoxide reductase A/B protein(CVF762) | Methionine sulphoxide reductase | *Neisseria gonorrhoeae* NCCP11945 | - |
| 98 | 40.31 | 1.00E-29 | orfM | putative deoxyribonucleotide triphosphate pyrophosphatase (CVF494) | LOS | *Haemophilus influenzae* PittGG | - |
| 99 | 44.12 | 1.80E-82 | phuS | hemin degrading factor (IA049) | direct heme uptake system | *Pseudomonas aeruginosa* PAO1 | - |

Note: “+” indicates present ; “-” indicates absent; “*”indicates different sources of VF species.
