# Supplementary material for: Whole-genome sequencing and analysis of Chryseobacterium arthrosphaerae from Rana nigromaculata
Source: BMC Microbiol. 2024 Mar 8;24:80. doi: 10.1186/s12866-024-03223-6 (PMC10921761; doi:10.1186/s12866-024-03223-6)
Supplement: Supplementary file 2 — Supplementary Material 2: Table S2: Differences in genes associated with antibiotic resisitance using Comprehensive Antibiotic Resisitance Database (CARD) between FS91703 and ED882-96 strains [file 12866_2024_3223_MOESM2_ESM.doc]

Table S2. Differences in genes associated with antibiotic resisitance using Comprehensive Antibiotic Resisitance Database (CARD) between FS91703 and ED882-96 strains

| No. | Resistance  Type | Description | strain FS91703 | strain ED882-96 |
| --- | --- | --- | --- | --- |
| 1 | catB8 | catB8 is a plasmid or integron-encoded variant of the cat gene found in *Klebsiella pneumoniae*, *Salmonella typhi* and *Pseudomonas aeruginosa.* | + | + |
| 2 | dfrE | dfrE is a chromosome-encoded dihydrofolate reductase found in *Enterococcus faecalis.* | + | + |
| 3 | farA | farA is the membrane fusion protein that is part of the farAB efflux pump. | + | + |
| 4 | sul2 | Sul2 is a sulfonamide resistant dihydropteroate synthase of Gram-negative bacteria, usually found on small plasmids. | + | — |
| 5 | IND-14 | IND-14 is a beta-lactamase found in *Escherichia coli.* | + | — |
| 6 | tetX | TetX is a flavin-dependent monooxygenase conferring resistance to tetracycline antibiotics. TetX hydroxylates position 11a of the tetraketide group thus inactivating the antibiotic. | + | — |
| 7 | Streptomyces | Sequence variants of *Streptomyces cinnamoneus* elongation factor Tu that confer resistance to elfamycin antibiotics. | + | — |
| 8 | catB6 | catB6 is a plasmid-encoded variant of the cat gene found in *Pseudomonas aeruginosa.* | + | — |
| 9 | catB2 | catB2 is a plasmid-encoded variant of the cat gene found in Escherichia coli, Salmonella enteritidis and Pasteurella multocida. | + | — |
| 10 | adeG | RND efflux transporter | + | — |
| 11 | thyA | Point mutations in the thymidylate synthetase thyA gene conferring resistance to paraaminosalicylic acid. | — | + |
| 12 | cmeB | CmeB is the inner membrane transporter the CmeABC multidrug efflux complex. | — | + |
| 13 | Pleuromutilin ABC | Pleuromutilin (Tiamulin) ABC efflux pump conferring resistance to pleuromutilin | — | + |
| 14 | rpoC | Point mutations that occurs in *Staphylococcus aureus* rpoC resulting in resistance to daptomycin | — | + |
| 15 | rpoB | Point mutations that occurs in *Escherichia coli* rpoB resulting in resistance to rifampicin | — | + |
| 16 | ArlR | ArlR is a response regulator that binds to the norA promoter to activate expression. ArlR must first be phosphorylated by ArlS. | — | + |
| 17 | kasA | Specific mutations on the *Mycobacterium tuberculosis* kasA gene resulting in lowered affinity of isoniazid, resulting in resistance | — | + |
| 18 | bcrA | bcrA is an ABC transporter found in Bacillus licheniformis that confers bacitracin resistance | — | + |
| 19 | fabG | fabG is a 3-oxoacyl-acyl carrier protein reductase involved in lipid metabolism and fatty acid biosynthesis. Thebacterial biocide Triclosan blocks the final reduction step in fatty acid elongation, inhibiting biosynthesis. Point mutations in fabG can confer resistance to Triclosan. | — | + |
| 20 | TLA-1 | TLA-1 is a beta-lactamase found in plasmids of clinical isolates of Escherichia coli strain R170 in Latin America. It preferentially hydrolyzed cephaloridine, cefotaxime,  cephalothin, benzylpenicillin, and ceftazidime. The enzyme was markedly inhibited by sulbactam, tazobactam, and clavulanic acid. | — | + |
| 21 | AbeS | AbeS in an efflux pump of the SMR family of transporters found in Acinetobacter baumannii. | — | + |
| 22 | arr-1 | arr-1 is a chromosome-encoded ribosyltransferase found in *Mycobacterium smegmatis.* | — | + |
| 23 | gyrA | Point mutation in Capnocytophaga gingivalis that decreases binding affinity of fluoroquinolone antibiotics to gyrA, thereby conferring resistance. | — | + |
| 24 | MexB | MexB is the inner membrane multidrug exporter of the efflux complex MexAB-OprM. | — | + |
| 25 | ykkD | ykkD is an SMR-type protein that is a subunit of the ykkCD efflux pump | — | + |
| 26 | dfrA3 | dfrA3 is an integron-encoded dihydrofolate reductase found in *Escherichia coli.* | — | + |
| 27 | VanG | D-Ala-D-Ala ligase homolog that can synthesize D-Ala-D-Ser, an alternative substrate for peptidoglycan synthesis that reduces vancomycin binding affinity | — | + |
| 28 | Tu | Sequence variants of *Enterococcus faecium* elongation factor Tu that confer resistance to GE2270A | — | + |

Note: “+” indicates present ; “-” indicates absent.
